# Influence of Synovial Fluid on Human Osteoblasts: An In Vitro Study

**DOI:** 10.1100/tsw.2007.282

**Published:** 2007-12-18

**Authors:** Thomas F. Fuchs, Wolf Petersen, Thomas Vordemvenne, Richard Stange, Michael Raschke, Jürgen R.J. Paletta

**Affiliations:** ^1^ Department of Trauma, Hand and Reconstructive Surgery, University Hospital Münster, Münster, Germany; ^2^ Department of Orthopaedics and Rheumatology, University Hospital, Marburg, Germany

**Keywords:** synovial fluid, osteoblast proliferation, osteoblast differentiation, bone healing, cruciate ligament reconstruction

## Abstract

Osseous graft healing at the tendon bone interface after anterior cruciate ligament (ACL) reconstruction is unsatisfactory in 10—25%, depending on the evaluation criteria or the kind of graft used for reconstruction. Mechanical as well as biological aspects are currently discussed. Since osteoblasts play an important role in the osseous integration of an ACL graft, we hypothesize that synovial fluid (SF), when entering the bone tunnel, has an inhibitory effect on osteoblasts. In order to verify this hypothesis, human osteoblasts (p3) were incubated in the presence of SF or partially purified SF. Proliferation was assayed using MTT or BrdU assay. Gene expression of osteoblast markers (alkaline phosphatase, collagen I, and osteocalcin) were determined by TaqMan analysis. In the control group, SF was exchanged by fetal calf serum (FCS). The results showed osteoblast proliferation in the presence of SF as well as in partially purified heat-pretreated synovial fluid. Native SF induced alkaline phosphatase and collagen I gene expression. No induction of the osteocalcin gene was observed in the experiment. These results were comparable to that obtained with FCS. These findings suggest that SF stimulated proliferation of osteoblasts in vitro. This effect is mediated, in part, by heat-stable components of SF. In addition, the expression of osteoblast marker genes alkaline phosphatase and collagen I, but not osteocalcin, was induced by SF. Therefore, problems associated with cruciate ligament reconstruction might be due to the inhibition of osteoblast differentiation. If so, this is not a specific attribute of SF, but also applies to serum.

## INTRODUCTION

Tendon grafts anchored within bony tunnels are used in a wide variety of orthopedic procedures. In the case of anterior cruciate ligament (ACL) reconstruction, osseous graft healing tends to result in implantation failure up to 25%, depending on the evaluation[1,2,3] or the kind of graft used for reconstruction (hamstring [HS][6,7] or achilles tendon allografts[8]). The mechanism of bone tunnel enlargement following ACL reconstruction is not yet clearly understood. Currently, mechanical factors, such as graft tunnel motion, stress deprivation of bone within the tunnel wall, improper graft tunnel placement, and aggressive rehabilitation[3,4,5,7,9], as well as biological factors, are discussed.

Clatworthy et al.[6] argued for a predominant biological component to bone tunnel enlargement. Biological factors thought to contribute to tunnel enlargement include a cytokine-mediated nonspecific inflammatory response, cell necrosis due to toxic products (ethylene oxide, metal), a foreign body immune response (allografts), and heat necrosis as a response to drilling[1,3,6,10].

It was also proposed that synovial fluid (SF), leaking into the bone tunnel, might be another possible reason[4,9,11,12]. In fact, during knee surgery and tunnel drilling, the graft as well as the tunnel comes in contact with SF. SF contains a cocktail of different substances that might have partially inhibiting effects on osseous healing of the graft.

In this study, the direct influence of SF on osteoblast proliferation and differentiation is investigated.

## MATERIALS AND METHODS

### Human Synovial Fluid

Human SF was obtained from patients undergoing arthroscopy of the knee after traumatic injury according to local ethics authorities. Surgery was done within 4 weeks after injury. Patient age ranged from 20 to 50 years (median: 31 years). In a provisional study, all single patient samples induced proliferation in osteoblasts in a similar degree as compared to control, and showed negligible differences as compared to the corresponding pool. On this account, for experiments, fluid samples of five patients with cruciate ligament rupture or rupture of menisci were pooled, in order to achieve suitable amounts of sample. All samples were frozen at –80°C for further analysis.

### Isolation of Human Osteoblast-Like Cells

Due to the fact that osteoblasts cannot be isolated from the same patient, cells were isolated from bone specimens obtained from the femoral neck of patients undergoing total hip replacement. These osteoblasts were used in order to examine mitogenic and osteogenic effects of SF. The patients’ ages ranged from 50 to 80 years (median: 71 years). Primary osteoblast cultures were obtained from bone specimens as described elsewhere[13]. Briefly, trabecular bone fragments were cut into pieces, thoroughly rinsed in phosphate buffered saline (PBS), and kept in MEM:HARMS F12 (1:1) (Biowest, Berlin, Germany) medium containing 10% FCS and antibiotics (100 U penicillin/ml, 100 µg streptomycin/ml) (Gibco, Life Technologies). Cultures were initiated in 75-cm² culture flasks (BD Biosciences, Bedford, MA) within 3 h and incubated at 37°C in a humidified atmosphere of 5% $CO_2$. Culture medium was replaced three times a week. Under these conditions, cells migrated out of the bone, forming a monolayer within 3–4 weeks. Primary cell layers were washed in PBS and detached with trypsin EDTA solution (Biowest, Berlin, Germany) and subcultured once in a 1:3 ratio. After growth to confluence, cells were released from culture dish as described above. Cells were either frozen at passage 1 or used for experiments in passage 3. Osteoblast-like phenotype was established by demonstrating alkaline phosphatase and osteocalcin expression.

### Proliferation Assay

Proliferation was assayed in 96 well plates. Cells were seeded at densities of 5000 cells/well in 200-µl MEM:HARMS F12 (1:1) medium containing 10% FCS or as indicated in the text. After attachment of the cells, medium was replaced by serum-free medium for 24 h before SF or pretreated SF was added (up to

10% final concentration) as described earlier[14,15]. Proliferation was analyzed with cell proliferation ELISA, BrdU (Roch, Indianapolis, IN) after incubation for 48 h at 37°C.

### Metabolic Activity

Metabolic activity was assayed with 3-(4,5-dimethylthiazol-2-yl)-2,5-diphenyltetrazolium bromide (MTT) using a commercially available kit (R&D Systems, Minneapolis, MN) according to the manufacturers instructions.

### Partial Purification of Synovial Fluid by Heat Treatment

Heat inactivation was performed by incubation of SF for 30 min at 90°C. After cooling, SF was centrifuged for 60 min at 2500 $\times g$ to remove denatured proteins and the supernatant (SFh) was retrieved for further processing.

### Total RNA Extraction and cDNA Synthesis

RNA was extracted from osteoblast cell layers after cultivation in the presence of SF or heat-pretreated synovial fluid (SFh) using RNeasy Mini Kit (Qiagen GmbH, Hilden, Germany) according to the manufacturer and quantified spectrometrically. Starting from 1-µg RNA, 20 µl cDNA were synthesized using Omniscript reverse transcriptase and oligo-dT primer in the presence of dNTP (Qiagen GmbH, Hilden, Germany).

### Quantitative RT-PCR

Reactions were performed and monitored using a ABI Prism 7700 Sequence Detection System (Applied Biosystems, Rotkreuz, Switzerland). The PCR 2X master mix was based on AmpliTAQ Gold DNA-Polymerase (Applied Biosystems, Rotkreuz, Switzerland). Genes of interest were analyzed in cDNA samples (5 µl for a total volume of 25 µl/reaction) using standard curve method (Perkin Elmer User Bulletin N. 2) Probes were labeled with 6-carboxy fluorescein (FAM) and TAMRA. Cycle temperatures and times were as previously described[17]. Primers and probes for human alkaline phosphatases (AP), collagen I (colI), osteocalcin (oc), and 18s rRNA were previously described[17,18] and purchased from (TIB Biomol, Berlin, Germany).

### Statistics

All values are presented as mean + SEM of three independent experiments. Differences between control group and SF group, or control group and SFh group were calculated using Mann-Whitney U Test with $p \leq 0.05$ considered to be significant.

## RESULTS

### Mitogenic and Metabolic Activities Induced by Synovial Fluid

First, we examined the ability of native SF to stimulate osteoblast proliferation as determined by BrdU assay, as well as metabolic activity determined by MTT assay. These activities were compared to that of untreated osteoblasts or osteoblasts grown in the presence of FCS as positive control. As shown in Fig. 1, the addition of SF to the culture medium resulted in a significant increase of proliferation ($p = 0.029$) and metabolic activity ($p = 0.008$). This increase was comparable to that induced by FCS.

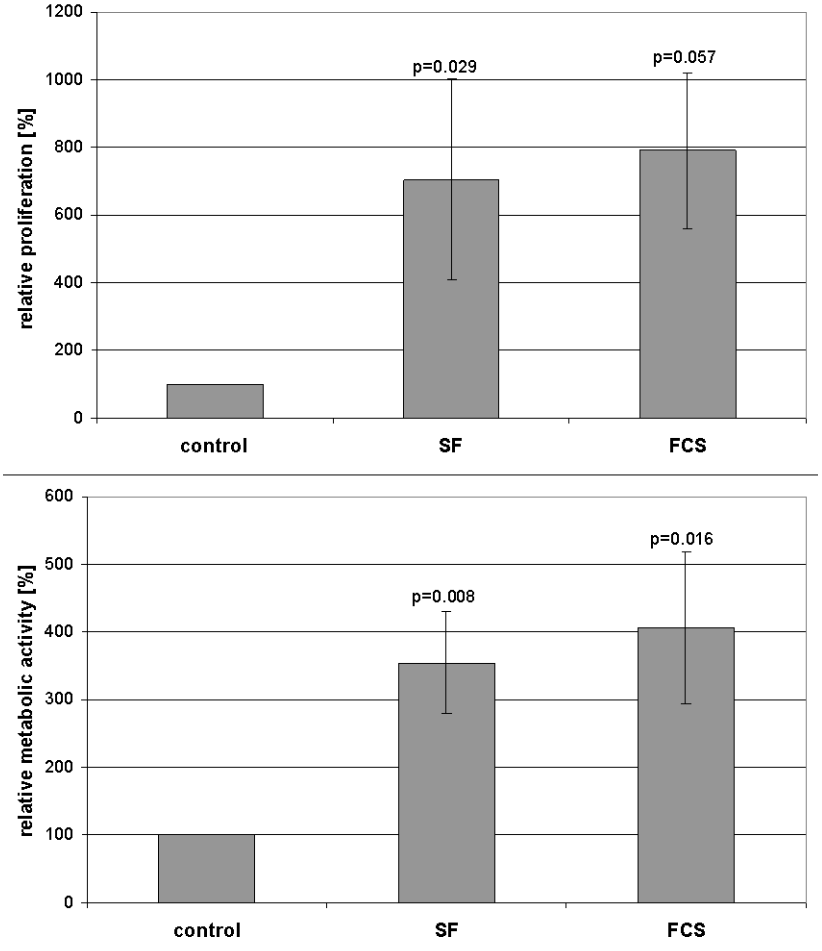

**FIGURE 1.** Mitogenic and metabolic activity SF on osteoblasts. Osteoblasts were seeded in a 96 well plate at a density of 5000 cells/well and exposed for 48 h to SF or FCS (40 µl/200 µl). The proliferation rate was determined with BrdU (top) and the metabolic activity with MTT assay (bottom) as described under Materials and Methods. Data represent mean ± SD from four independent determinations done in triplicate.

In order to characterize the mitogenic activity of SF, partial purification was performed by heat pretreatment of three independent pools of SF (five patients each). SFh had a mitogenic activity of 259 ± 42% (determined by BrdU [$p = 0.029$]) and 253 ± 10% (determined by MTT [$p = 0.008$]) as compared to untreated control (Fig. 2). This was a decrease by approximately 50% compared to native SF.

## Influence of Synovial Fluid on Expression of Bone-Related Proteins

Although osteoblast proliferation is critical for the formation of new bone, it may be argued that mitogeniticity alone is not sufficient for supporting effective bone regeneration during cruciate ligament reconstruction. Therefore, we next sought to examine directly the influence of SF and SFh on the

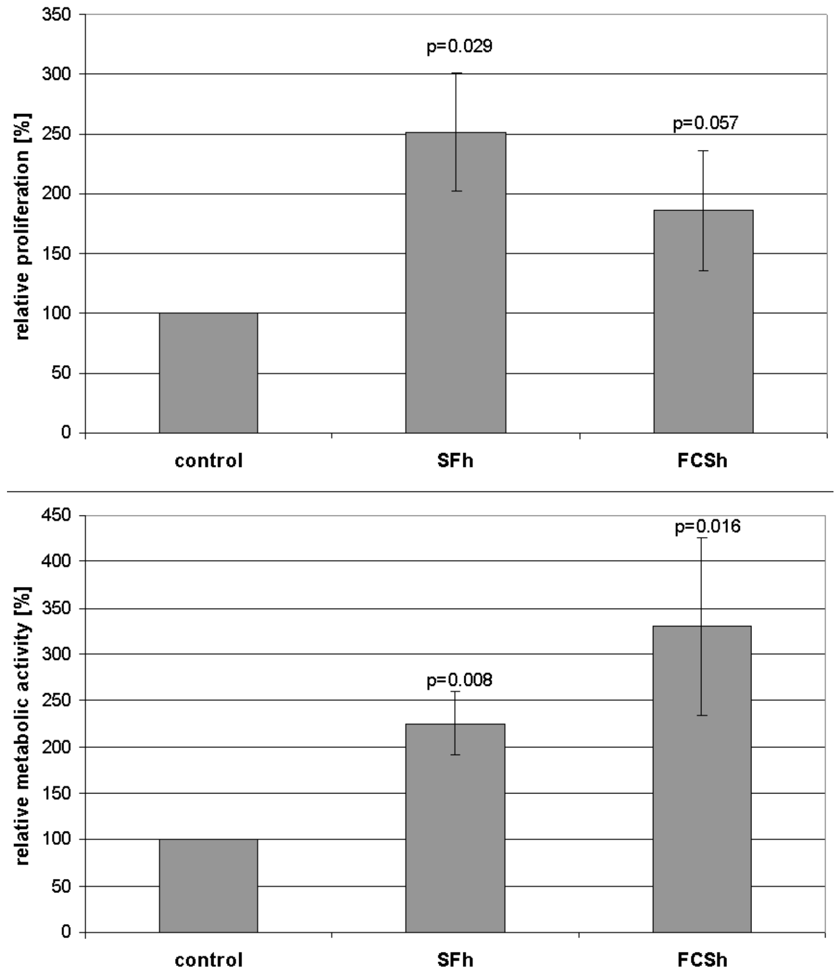

**FIGURE 2**. Mitogenic and metabolic activity of SFh on osteoblasts. Osteoblasts were seeded in a 96 well plate at a density of 5000 cells/well and exposed for 48 h to SFh or FCS (40 µl/200 µl). The proliferation rate was determined with BrdU (top) and the metabolic activity with MTT assay (bottom) as described under Materials and Methods. Data represents mean ± SD from four independent determinations done in triplicate.

differentiation of osteoblasts *in vitro*. Gene expression of bone-specific alkaline phosphatase, collagen I, and osteocalcin were analyzed using real-time PCR. Cells stimulated with SF, SFh, FCS, and heat-pretreated FCS (FCSh) (20% each) were cultured at a density of $1 \times 10^5$ cells/well in 24 well plates over a period of 24 h. Basal osteoblast gene expression was used as control.

As shown in Fig. 3A, SF significantly induces alkaline phosphatase gene expression (178 ± 38%; $p$ = 0.029). This effect was unaffected by the heat pretreatment of the SF. The effect of FCS on gene expression of alkaline phosphatase of osteoblasts was alleviated compared to that of SF, but did not reach significant levels (150 ± 60%; $p$ = 0.629).

Collagen I gene expression (Fig. 3B) was significantly enhanced when osteoblasts were cultured in the presence of SF (193 ± 14%; $p$ = 0.029) or FCS (183 ± 40%; $p$ = 0.057). Minor induction of the collagen I gene was observed when osteoblasts were cultured in the presence of SFh or FCSh, but this effect was not significant.

On the other hand, osteocalcin gene expression (Fig. 3C) was reduced when osteoblasts were cultured in the presence of SF (54 ± 29%; $p$ = 0.057) or FCS (54 ± 19%; $p$ = 0.029).

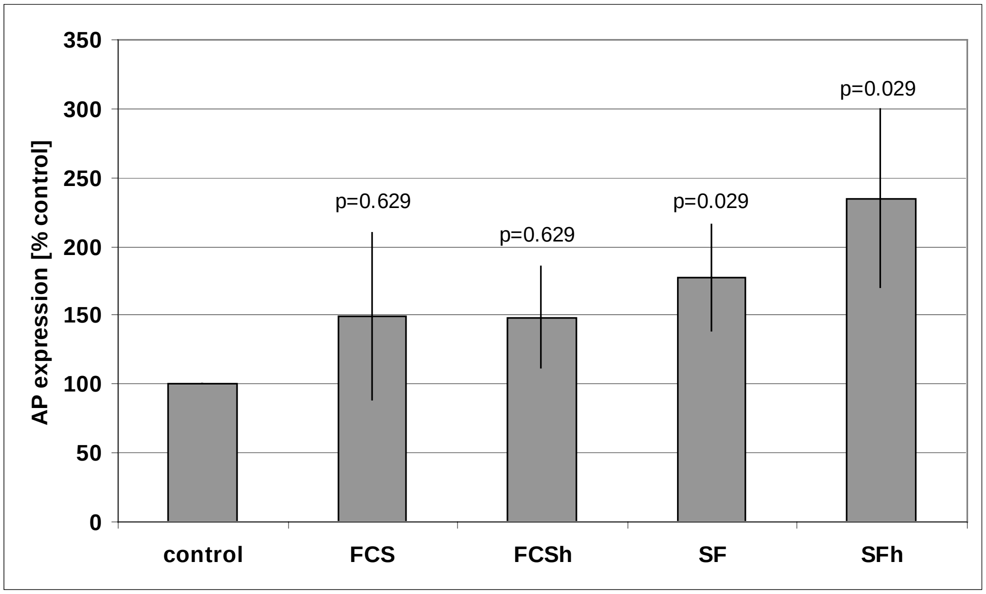

A

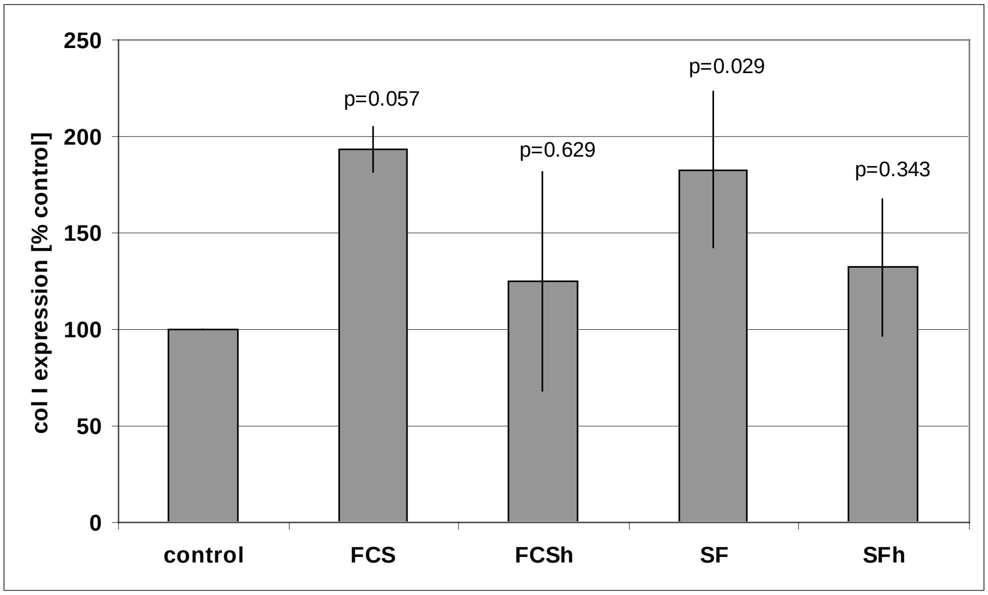

B

**FIGURE 3.** Influence of SF on expression of bone-related proteins. Human osteoblasts were cultured at a density of $1 \times 10^5$ cells/well in 24 well plates in the presence of SF, SFh, FCS, and FCSh over a time period of 24 h. Gene expression of alkaline phosphathase (A), collagen I (B), and osteocalcin (C) was measured relative to 18s expression. Values are shown as mean ± SEM of three independent examinations

## DISCUSSION

The effective osseous integration of a tendon graft depends on balanced activity of bone-forming osteoblasts, bone-degrading osteoclasts, as well as tendon fibroblasts.

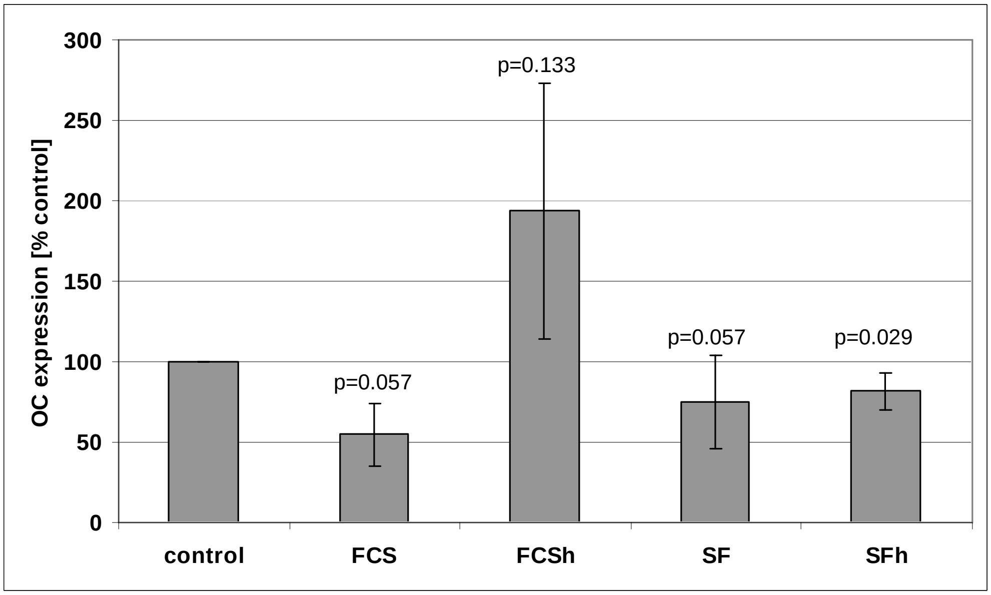

**FIGURE 3C**

In this study, the hypothesis was elucidated whether SF, when entering the bone tunnel, is inhibitory to osteoblasts.

Based on earlier studies showing that SF contains growth factors[19], proinflammatory cytokines[20], human platelets[21], inhibitors of osteoblast proliferation[22], and hyaluronate[23], it is likely that the fluid mediates activating as well as inhibitory attributes towards osteoblasts. To our knowledge, this is the first study on cumulative effects of SF on proliferation and differentiation of human osteoblasts.

Our results, based on two independent methods, showed a mitogenic effect of SF on osteoblasts, which was comparable to that of identical amounts of serum. So far, our *in vitro* studies gave no evidence that SF is inhibitory to osteoblast proliferation.

These findings are supported by Nickerson et al.[15], who showed that SF stimulates ligament fibroblast proliferation *in vitro*. On the other hand, Andrish and Holmes[24] found inhibitory effects of SF on cell proliferation. These conflicting results were not restricted to ligament fibroblasts. Andersson et al.[14] showed that the mitogenic activity towards osteoblasts depends on the origin of SF. Fluids obtained from patients with loose hip arthroplasties were inhibitory to osteoblast proliferation, whereas fluids from patients with osteoarthritis induced proliferation in osteoblasts. We interpret these conflicting publications as alteration of the biochemical consistence of SF, resulting in differential mitogeniticity towards osteoblasts or fibroblasts. Therefore, we cannot exclude that the operative procedure of ACL reconstruction itself can possibly influence the consistence of SF, resulting in an inhibition of osteoblast proliferation. Nevertheless, before ACL reconstruction, native SF is mitogenic towards osteoblasts. In extension of the results of Andersson et al.[14], we could show that SF possesses the capacity to induce alkaline phosphatase and collagen I expression of osteoblasts. Both are crucial for the early osteoblast function. In contrast, osteocalcin gene expression, necessary for calcification and late-stage differentiation, was diminished. The latter is necessary for bone formation. Therefore, a reduced capacity in osteoblast differentiation might also result in a reduced healing capacity of cruciate ligament transplantation. This indicates that osteoinductivity is not a common property of SF and inhibits osteoblast differentiation (defined as osteocalcin expression). Therefore, problems associated with cruciate ligament reconstruction might be due to the inhibition of osteoblast differentiation. If so, this is not a specific attribute of SF, but also applies to serum.

In order to get more knowledge of the mitogenes in SF, partial purification by heat pretreatment was performed. The results gave evidence that aside from common polypeptide growth factors or cytokines, the fluid possesses a heat-stable mitogenic activity. This contributes at least up to 50% to the total mitogenic activity. Previous studies[16] with endothelial cells have shown that heat-stable mitogenic activity was mediated by HDL-associated phospholipids like sphingosine 1 phosphate (S1P). Indeed, SF contains HDL (data not shown) or, in case of trauma, platelets containing S1P. Therefore, we speculate that the effect described here is accelerated by HDL-associated or platelet-released S1P, which is known to stimulate osteoblast proliferation[25,26,27]. Ongoing studies using alkaline phosphatase digestion or pharmacological inhibitors, as well as receptor analysis, support the hypothesis.

## ACKNOWLEDGMENT

We gratefully acknowledge the excellent technical assistance of Simone Niehues.
